# The cyclin-dependent kinase inhibitor p57^Kip2^ is epigenetically regulated in carboplatin resistance and results in collateral sensitivity to the CDK inhibitor seliciclib in ovarian cancer

**DOI:** 10.1038/bjc.2011.566

**Published:** 2012-01-10

**Authors:** H M Coley, N A M Safuwan, P Chivers, E Papacharalbous, T Giannopoulos, S Butler-Manuel, K Madhuri, D P Lovell, T Crook

**Affiliations:** 1 Faculty of Health and Medical Sciences, University of Surrey, Guildford, Surrey GU2 7XH, UK; 2 Section of Gynaecological Oncology, Royal Surrey County Hospital, Guildford, Surrey GU2 7XX, UK; 3 St George's University of London, Cranmer Terrace, London SW17 ORE, UK; 4 Charing Cross Hospital & Imperial College, London, UK

**Keywords:** ovarian cancer, p57^Kip2^, epigenetically silenced, carboplatin resistance, seliciclib

## Abstract

**Background:**

Carboplatin remains a first-line agent in the management of epithelial ovarian cancer (EOC). Unfortunately, platinum-resistant disease ultimately occurs in most patients. Using a novel EOC cell line with acquired resistance to carboplatin: PEO1CarbR, genome-wide micro-array profiling identified the cyclin-dependent kinase inhibitor *p57*^*Kip2*^ as specifically downregulated in carboplatin resistance. Presently, we describe confirmation of these preliminary data with a variety of approaches.

**Methods:**

Cytotoxicity testing (MTT) and cell cycle blockade assessed drug responsiveness. Methylation specific PCR and pyrosequencing identified sites of promoter methylation in *p57*^*Kip2*^. siRNA to *p57*^*Kip2*^ was used to look at the changes in apoptosis of carboplatin treated EOC cells. EOC tissues (20 cases) were assessed for mRNA levels of p57^Kip2^.

**Results:**

Carboplatin resistance was reversed using 5-aza-cytidine *in vitro*. Promoter methylation sites and preferential sensitivity to seliciclib were seen in PEO1CarbR cells. Silencing *p57*^*Kip*^*2* decreased the apoptotic response to the effects of platinum but produced sensitisation to seliciclib. EOC biopsies indicated an association of high levels of p57^Kip2^mRNA with complete responses to chemotherapy and improved outcome.

**Conclusion:**

We conclude that p57^Kip2^ is a candidate biomarker of platinum sensitivity/resistance in EOC and such cases may show preferential response to the cyclin-dependent kinase inhibitor seliciclib.

p57^Kip2^ is a member of the CIP/KIP family of cyclin-dependent kinase inhibitors (CDKI). Each member of this family possesses a conserved domain at their N-terminus called the CDK binding-inhibitory domain, unlike INK4 family members, which are characterised by four tandem repeats of an ankyrin motif (Beretta *et al*, 2005). In humans, the *p57*^*KIP2*^ gene is situated on chromosome 11p15.5 in a cluster of imprinted genes such as *IGF-II* and *H19* (Beretta *et al*, 2005; Kim *et al*, 2005; Roeb *et al*, 2007). The gene is associated with a high frequency of heterozygosity loss in human cancers and functions as a CDKI by binding to cyclin E–CDK2 complexes, leading to inhibition of G1 to S phase progression. Cyclin E–CDK2 is essential for pRb phosphorylation and subsequent gene transcription by E2F. The action of p57^Kip2^ on the cyclin E–CDK2 complex promotes cell-cycle exit and cell differentiation. Differentiation signals have been shown to induce accumulation of *p57*^*KIP2*^ mRNA (Roeb *et al*, 2007). P57^Kip2^ has a major role in embryogenesis predominantly in early organogenesis, with ubiquitous expression in the whole embryo, which decreases along with the increased embryo development (Beretta *et al*, 2005). It is the only CDKI associated with normal embryonic development, with loss of expression in mouse embryos leading to defective proliferation in lens and cartilage, and developmental defects in several tissues and increased postnatal lethality (Zhang *et al*, 1998). Deficiency of p57^Kip2^ in the mouse leads to organomegaly with abdominal wall defects, increased apoptosis, delayed differentiation during development, immaturity of some tissues, severe growth retardation and placentomegaly (Kim *et al*, 2005). Loss of functional p57^Kip2^ is also a common feature of rhabdomyosarcoma, even though it is not a dominantly acting transforming event (Roeb *et al*, 2007) and has also been reported for breast cancer, suggesting its role as a putative tumour suppressor gene (Larson *et al*, 2008).

Cell-cycle dysregulation is a feature of many cancers, including epithelial ovarian cancer (EOC) (D’Andrilli *et al*, 2004). Moreover, dysregulation of cell-cycle components seems specific for certain histological types of EOC. Serous EOC is the most commonly observed subtype of this disease with a poorly understood molecular aetiology. Overexpression of the CDKI p21^Cip1/Waf11^ has previously been shown to be associated with early-stage serous tumours. Overexpression of p53 and cyclin E and reduced expression of p27^Kip1^ and p21^Cip1/Waf1^ is associated with increasing tumour grade (Bali *et al*, 2004). There are isolated reports indicating lower p57^Kip2^ protein level in EOC tissue from patients with a poor prognosis (Rosenberg *et al*, 2001).

Previous work in our laboratory has involved the generation of novel drug resistant models of EOC as part of an ongoing programme to understand the basis for chemoresistance in this disease. An initial microarray analysis of the human PEO1 human EOC line and novel drug resistant variants revealed low levels of the gene *p57*^*Kip2*^ as a consequence of carboplatin resistance. Further, methylation reversal using demethylating agents suggested a mechanism of gene silencing underlying the reduced expression of p57^Kip2^ seen in carboplatin resistant cells. We have further examined this initial finding using quantitative PCR (qPCR) analysis, western immunoblotting of the same cell lines, chemosensitivity testing and analysis of freshly extracted ovarian cancer tissue for mRNA levels of p57^Kip2^ with assessment of clinico-pathological features.

## Materials and methods

### Cell lines and culture conditions

The human ovarian cancer cell line PEO1 (kindly provided by Dr F Balkwill, formerly of ICRF, London, UK) was grown in RPMI-1640 medium (Sigma-Aldrich, Poole, UK) supplemented with 10% heat-inactivated foetal calf serum (FCS) and 2 mM Glutamax. All cell culture reagents were obtained from Sigma (Poole, UK) unless stated otherwise. The ovarian carcinoma cell line PEO1 (parental) was cultured as a monolayer in RPMI-1640 medium and supplemented with 10% FCS (heat inactivated; Invitrogen, Paisley, UK) and maintained at low-passage number. The drug resistant variants PEO1CarbR (carboplatin resistant) and PEO1CisR (cisplatin) were derived by step-wise incubation of the chemotherapeutic agent over a number of weeks. The maintenance dose for PEO1CisR was 1 *μ*M cisplatin and for PEO1CarbR 2 *μ*M carboplatin. These cell lines have previously been described (Coley *et al*, 2006).

### Cytotoxic drugs

Cisplatin and carboplatin (Sigma) was dissolved as a stock solution in sterile 0.9% saline and stored at −20°C until use. 5′-aza-2′ deoxycytidine (obtained from Sigma-Aldrich) was made up in sterile distiled water and stored at −20°C in aliquots before use in experiments. Seliciclib was obtained from Cyclacel Ltd (Dundee, Scotland, UK) and from Selleck Chemicals (Houston, TX, USA), made up in DMSO and stored as frozen aliquots.

### Drug treatment of cells for methylation reversal

**Figure 1:**
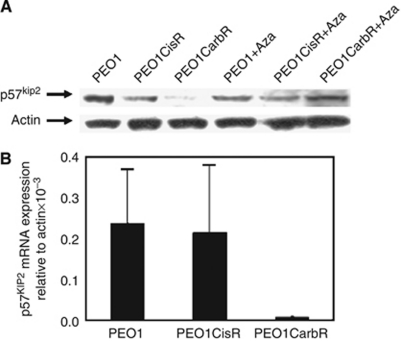
(**A**) Western immunoblotting of PEO1 cell lines without and with 5-aza-2-deoxy-cytidine/trichostatin A treatment (+aza) using SDS–PAGE gels (NOVEX system, Invitrogen) followed by western transfer onto PVDF membranes. Membranes were probed with a primary rabbit polyclonal antibody to p57^kip2^ sc-8298 (clone H-91) (Santa Cruz, distributors Autogen Bioclear, Calne, UK). Membranes were stripped and reprobed for actin as a loading control. (**B**) Expression levels of mRNA p57^Kip2^ in the PEO1 panel of cell lines obtained qPCR with SyBr green and actin as the internal control. Data shown are the mean (and s.d.) for at least three repeat experiments.

For the experiments that looked at the effects of demethylation on platinum sensitivity and protein expression levels of p57^Kip2^, prolonged exposure of demethylating drug was used for all cell lines. The cultures were grown up for four to five passages in the presence of 5′-aza-cytidine at a dose of 1 *μ*M for 5 days followed by Trichostatin A 200 nM for 2 days (modified from the protocol described by Syed *et al*, 2006), as repeated dosing with higher doses gave rise to pronounced growth arrest and loss of viability, particularly evident for the parental cell line. Note that the carboplatin-resistant cells were not treated with their maintenance dose of carboplatin during this time period. These cells were then used in the MTT assay as shown in Figure 1C.

### Cytotoxicity testing using the MTT assay

Cells for cytotoxicity testing were seeded into 96-well plates and allowed to attach and equilibrate for 24 h in a humidifying incubator at 37°C and 5% CO_2_. They were then treated with various concentrations of drug over two to three log orders of concentration and left for 72 h to allow for approximately three to four doublings (∼10-fold increase in control cell number) over the duration of the drug exposure. Drugs were diluted in tissue culture medium containing 10% FCS. Control cell wells were treated with an aliquot of drug-free tissue culture medium. Cell viability was determined by addition of 4 × 10^−4^ mg MTT (thiazoyl blue tetrazolium bromide; Sigma-Aldrich) for 4 h at 37°C. Wells were aspirated to remove the medium and the resulting formazan crystals solubilised in 200 *μ*l DMSO and the absorbance read at 570 nM. The absorbance of the formazan product obtained for drug-treated cells was calculated as a fraction of that for the untreated control wells. Growth curves were constructed using PRISM software (GraphPad Software Inc., La Jolla, CA, USA) and results expressed as IC_50_ values, that is, dose of drug causing a 50% reduction in cell viability.

### Assessment of cell-cycle perturbations following seliciclib treatment of ovarian cancer cells

Cells were seeded in culture flasks, and incubated for 24 h, before treatment with seliciclib (25 *μ*M) for 48 h. This corresponded to the cells being in early-exponential phase of growth, ∼30–40% confluent. At the end of the treatment period, cells were removed by trypsinisation, centrifuged and the pelleted cells were washed in PBS and resuspended in ice-cold 70% ethanol in PBS (added slowly whilst vortex mixing). Samples were left at 4°C for at least 24 h. After washing in PBS, cells were stained in 5 *μ*g ml^−1^ propidium iodide (PI) and 1 mg ml^−1^ ribonuclease A for at least 30 min at 37°C in the dark. Fluorescence at 575 nm (FL3) was measured on a Beckman-Coulter Epics XL flow cytometer. Cell-cycle phases were evaluated using the Multi-Cycle software program (Beckman Coulter, High Wycombe, UK).

### Assessment of apoptotic response following silencing of p57^KIP2^

Cells were cultured in six-well plates before transfection with siRNA against *p57*^*KIP2*^ (obtained from Dharmacon, ThermoFisher, Epsom, UK). The process was carried out according to manufacturer's instructions and cells were left to grow for 72 h, using appropriate controls. After this time, cells were harvested and seeded into 25-cm^2^ flasks to ∼40% confluence. After attachment, the cells were then treated with various doses of carboplatin and with seliciclib for a period of 48 h. The doses of drug are indicated in the appropriate legend. Cells were then harvested and subjected to the Annexin V assay combining with PI according to manufacturer's instructions (CN Biosciences, Beeston, UK). Samples were then subjected to flow cytometry, using FL1 for FITC conjugated Annexin and FL3 for PI on the Coulter EPICS flow cytometer.

### DNA isolation

DNA was extracted from cell lines with and without treatment with the demethylating agent 5-Aza-2′deoxy-cytidine 5 *μ*M and with Trichostatin A 200 nM for successive passages, as described above. This was carried out using the GenElute genomic DNA kit (Sigma-Aldrich) according to manufacturer's instructions.

### Methylation specific PCR (MSP)

Methylation specific PCR was carried out using 1 μg of genomic DNA with bisulphite modification using the EZ DNA methylation kit (Zymo Research; obtained from Cambridge Biosciences, Cambridge, UK) according to manufacturer's instructions. For each assay set up a control universal methylated DNA standard (Zymo Research; Cambridge Biosciences) was also taken through the process to ensure that bisulphite conversion was complete. Primers were designed using the MethPrime program (http://itsa.ucsf.edu/?urolab/methprimer). The PCR for the *p57*^*KIP2*^ CpG island was performed using HotStart Mastermix (Qiagen Ltd, Crawley, UK) using a thermal cycler with the following protocol: 95°C for 15 min; 40 cycles of: 95°C for 30 s, 52°C for 30 s, 72°C for 30 s, for the U (unmethylated sequence) and 95°C for 15 min; 40 cycles of 95°C for 30 s, 59°C for 30 s and 72°C for 30 s for the M (methylated sequence).

The sequences for the primers (U; M) were as follows:

Left M primer: TGTAGTTCGCGGTTTAGTTTTTC-3′
Right M primer: ATCCACGATAAAACGTCTTATCG-3′
Product size: 114 bp
Left U primer: TTTGTAGTTTGTGGTTTAGTTTTTT-3′
Right U primer: ACATCCACAATAAAACATCTTATCA-3′
Product size: 118 bp

PCR reactions were carried out on at least three separate occasions and the products then visualised using 0.8% agarose separation with TAE buffer with ethidium bromide staining and transillumination.

### DNA methylation analysis using pyrosequencing

2 *μ*g of DNA was bisulfite-converted using Qiagen Epitect Bisulfite Conversion kit and following the manufacturer's protocol. The bisulfite-converted DNA was eluted from the column to 40 *μ*l final volume. For pyrosequencing PCR, 2 *μ*l of the bisulfite-converted DNA was used in a final 50 *μ*l reaction volume. The PCR cycle used was: 95°C for 15 min; 50 cycles of: 95°C for 30 s, 60°C for 30 s, 72°C for 30 s and 72°C for 5 min. The pyrosequencing was carried out using the Biotage PSQ96MA pyrosequencer (Biotage AB, Uppsala, Sweden). The primers used were:

Forward primer: 5′-AGGGGAGGGTTGATAGTTA-3′

Biotinylated reverse primer: Bio5′-CTAAAATTACCACTTCCAACAAAACATACC-3′

Sequencing primer: 5′-GGGGAGGGTTGATAGTTAT-3′

The primers were designed using Pyromark Assay Design Software 2.0 (Qiagen Ltd) to match the region targeted by MSP primers. The primer design was targeted from 1301 to 1700 bases of the UCSC Genome Browser March 2006 (NCBI36/hg18) annotated CpG island191. The final primers selected had a score of 77 and were passed through the software quality control. The PCR product size was 117 bp. The sequencing primer covered 17 target polymorphisms/CpG sites.

### Western immunoblotting

Whole-cell lysates were obtained by trypsinising the monolayer of adherent cells and washing with PBS at 4°C. Cell pellets were then subjected to osmotic rupture in hypotonic detergent based buffer (1 mM PMSF, NaVO_4_, aprotinin and leupeptin as protease inhibitors, 150 mM NaCl, in 50 mM Tris buffer, 0.2% SDS, 1% NP-40, pH 7.5) and 50 *μ*g of protein per sample electrophoresed on SDS–PAGE gels with subsequent transfer blotting. Membranes were incubated overnight at 4°C with primary antibody. After washing, membranes were incubated with a secondary horseradish peroxidase-linked appropriate species antibody preparation at room temperature for 1 h followed by chemiluminescence for visualisation. Following the probing of each membrane with the primary antibody, the membrane was stripped and reprobed using an actin antibody to act as a loading control.

### Clinical samples and correlation with outcome

Clinical cases of EOC were obtained by informed consent by the Section of Gynaecological Oncology and by local ethical committee approval, St Luke's Cancer Centre, Royal Surrey County Hospital (Guildford, Surrey, UK). In each case, the diagnosis and presence of adequate tumour representation in the specimen was confirmed by histopathological analysis. The histological classification for the samples was shown to be: serous (13 cases); clear cell, including those with mixed endometrioid histology (1+2 cases); mixed Mullerian tumour (1 case) and endometrioid (2 cases). As all these women underwent primary surgery at the time of sample collection, they then received six cycles of postoperative chemotherapy, which included combination of carboplatin and paclitaxel or a single agent carboplatin only depending on their performance status. Follow-up and mature data including response to treatment, disease free interval and overall survival is also recorded. Assessment of response to treatment included clinical examinations, serum CA 125 levels and computerised tomography scans when indicated. Patients were classified as complete, partial and non-responders based on the above three criteria, which in turn depended on the bulk of residual disease following surgery. Minimal residual disease with normal CA 125 indicated a complete response (CR). A measurable reduction in disease by ⩾30% on clinical and radiological examination constituted a partial response (PR). Progression on treatment or relapse within 6 months suggested a non-response.

### Collection and RNA extraction of ovarian cancer biopsies

Tissue was obtained at first laparotomy before chemotherapy and then rapidly frozen at −80°C for short-term storage before extraction. The frozen biopsies in RNALater solution (Qiagen Ltd) were thawed on ice and preservation fluid aspirated. RNA was isolated using the RNeasy method (mini-column procedure; Qiagen Ltd) with sample lysis using the TissueLyser (Qiagen Ltd). The quality of the resulting RNA was checked with an Agilent 2100 Bioanalyser using an RNA NanoLabchip according to the manufacturer's instructions (Agilent Technologies UK Ltd, Stockport, Cheshire, UK). The RNA was diluted with RNase free water and quantified using the Nanodrop spectrophotometer (Nanodrop Technologies Inc., Fisher Scientific, Loughborough, UK). Following verification of the quality of RNA extracted, 19 samples were deemed suitable for further analysis.

### Real-time PCR for p57^Kip2^ in ovarian cancer cell lines and clinical biopsies of EOC

cDNA was synthesised from 2 *μ*g of total RNA. PCR was performed in an ABI PRISM 7700 Sequence Detection System (Applied Biosystems, Life Technologies Ltd, Paisley, UK) using the dye SyberGreen qPCR mastermix (Qiagen Ltd). Primers for p57^Kip2^ were obtained from SuperArray (Tebu-Bio, Leicester, UK) with gene identification of *CDKN1C* (NM_000076). Primers for actin were obtained from Invitrogen. Sequences were: forward: 5′-GCATCCACGAAACTACCTTC-3′ reverse: 5′-CAGGAGGAGCAATGATCTTG-3′.

For qPCR reactions, 1.5 mM MgCl_2_ was used for all primers (p57^Kip2^ and actin) and the cycling conditions used were: 40 cycles at 95°C for 30 s, 58°C for 30 s and 72°C for 40 s. Fold inductions were calculated using the formula 2^−(ΔΔCt)^, where ΔΔCt is the ΔCt _(p57_^Kip2^_)_-ΔCt _(actin)_ and Ct is the cycle at which the threshold is crossed.

### Statistical analysis

Expression levels of mRNA for p57^Kip2^ of the groups of samples split into categories, which can be described as CR, PR and NR (no response) were assessed using a one-way ANOVA using Minitab. The CR group was compared with the PR and NR group combined because of the small sample size for the latter group. The survival times of the different groups were compared using a log rank test using SPSS Version 12 software (SPSS Inc., Chicago, IL, USA). Other *t*-tests were performed to compare IC_50_ doses obtained from dose-response curves and these are quoted as appropriate.

## Results

### Cell lines

We have previously derived a novel cell line with acquired resistance to carboplatin, PEO1CarbR, from the PEO1 parental cell line, as described (Coley *et al*, 2006). We have also included the PEO1CisR (cisplatin-resistant variant) for comparison in some experiments.

### p57^Kip2^ is epigenetically downregulated in carboplatin-resistant EOC cells

To seek genes differentially expressed between parental and drug resistant cell lines, we performed an initial microarray expression analysis screen (data not shown). We observed a marked reduction in *p57*^*Kip2*^ expression in the PEO1CarbR cells and thus set about confirming the protein expression and mRNA levels. We performed qPCR and western blot analysis of p57^Kip2^ expression in the PEO1 parental, cisplatin-resistant and carboplatin-resistant cell lines. These experiments showed that p57^Kip2^ mRNA (and protein) levels were greatly reduced in PEO1CarboR relative to the parental PEO1 cells (Figure 1A and B). For PEO1CisR cells we saw that expression of p57^kip2^ levels fluctuated but generally the levels were similar or higher than the parental PEO1 cell line.

### Demethylation increases p57^kip2^ expression in carboplatin-resistant cells

The initial microarray assay incorporated a pre-treatment step with aza-cytidine and zebularine (both demethylating agents) for comparison of gene expression levels with untreated cells. We saw evidence of unmasking of the *p57*^*kip2*^ gene expression levels In order to corroborate this initial observation we tested whether treatment with 5-aza-2′deoxy-cytidine restored p57^kip2^ protein expression levels in the cell line panel. We demonstrated that at the protein level demethylation treatment can unmask the expression of p57^kip2^ in the PEO1CarbR line, with no significant effect being seen for the parental or PEO1CisR line, as shown in Figure 1A.

### Drug sensitivity of PEO1 lines and effects of demethylation treatment to carboplatin and seliciclib

**Figure 2:**
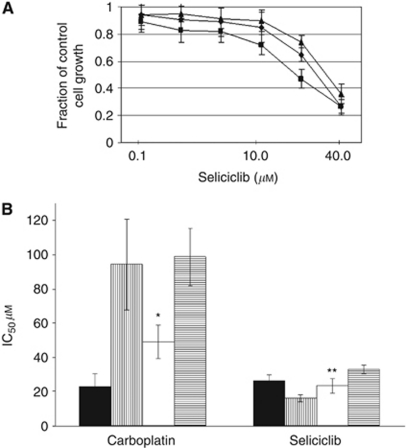
(**A**) Dose-response curves for PEO1 (circle), PEO1CisR (triangle) and PEO1CarbR (square) treated with seliciclib (*n*=>3, with the s.d. shown for each data point (drug concentration used) as error bars). (**B**) IC_50_ data obtained from dose-response curves (using MTT) with continuous drug exposure for PEO1 parental (black), PEO1CarbR (vertical shading), PEO1CarbR demethylated (white) and PEO1CisR (horizontal shading) for carboplatin and for seliciclib treatments. All experiments were carried out on at least three separate occasions and data show the IC_50_ as mean with s.d. as error bars, *P*-values obtained for comparison of IC_50_ values for PEO1CarbR cells *versus* PEO1CarbR-demethylated cells were 0.0055 (^*^) for carboplatin and 0.016 (^**^) for seliciclib (paired *t*-test). For carboplatin the resistance factors obtained for PEO1CarbR was 4.2 and for PEO1CarbR-demethylated was 2.2.

Given the association of p57^kip2^ with CDK2 and cyclin E we considered the use of a selective CDK2 inhibitor drug as a possible treatment strategy in p57^kip2^ deficient cells. Using the MTT assay with continuous drug exposure (72 h) we saw that PEO1CarbR cells showed preferential sensitisation to seliciclib in comparison with PEO1 parental and PEO1CisR cells based on IC_50_ values: PEO1 26.4 *μ*M (−/+ 3.51), PEO1CisR 33.0 *μ*M (−/+ 2.71) and PEO1CarbR 16.0 *μ*M (−/+ 2.06), *P*<0.005 (using a one-tailed paired test) comparing IC_50_ value for PEO1CarbR with either PEO1 or PEO1CisR. Dose-response curves for the PEO1 lines treated with seliciclib are shown in Figure 2A.

We then used a similar epigenetic reversal treatment protocol as in western blotting experiments (Figure 1A) and assessed drug sensitivity following this. 5-Aza-2′deoxy-cytidine had a negligible effect on sensitivity to either carboplatin or seliciclib in parental PEO1 cells (IC_50_ values not changed by >10%, data not shown). For the time period we used the epigenetic reversal protocol (*c*. 4 weeks), the drug resistant variant maintained a drug resistant phenotype in parallel cultures, under standard culture conditions (in the absence of the demethylating agent), indicating that the resistance phenotype was stable for the study period in the absence of the inducing agent (carboplatin). As depicted in Figure 2B, the IC_50_ values (*μ*M) following treatment of cells with continuous exposure to carboplatin were as follows giving the mean and (s.d.) for at least three separate replicate analyses: PEO1 22.7 (7.8), PEO1CarbR 94.3 (26.4) and PEO1CisR 98.7 (16.7). In addition, for the PEO1CarbR-demethylated cells the level of carboplatin resistance was reduced with a shift in IC_50_ down to 49.2 *μ*M (*P*=0.0055 using a one-tailed paired test). In the same experiments we saw that the level of seliciclib sensitivity was reduced in the PEO1CarbR-demethylated cells going from 16.0 to 23.3 *μ*M (*P*=0.016 using a one-tailed paired test) following unmasking of p57^kip2^.

### Carboplatin–resistance associated with p57^Kip2^ silencing gives rise to collateral sensitivity to the CDKI seliciclib, which is shown by a prolonged S-phase

**Figure 3:**
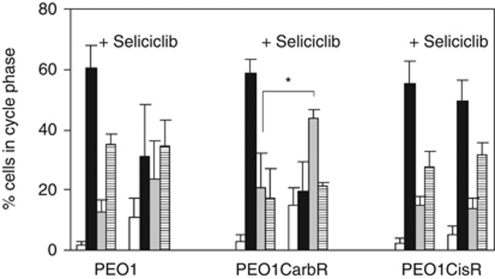
Cell-cycle analysis for PEO1 cell lines treated with seliciclib (25 *μ*M) for 48 h. Key: SubG1, white; G1, black; S-phase, grey; G2M, horizontal line. *N*=3 separate experiments. Cells were stained with PI (see Materials and Methods) and analysed used a Coulter Epics flow cytometer with data analysis using MultiCycle software (Beckman Corp.). The S-phase for treated PEO1CarbR cells *versus* control gave a *P*-value of 0.03, indicated as ^*^.

Treating asynchronous cultures of the PEO1 panel of cell lines with a 25 *μ*M dose of seliciclib gave rise to cell-cycle perturbations, as seen in Figure 3, with the biggest differences being seen for PEO1CarbR in terms of the decrease in G1 (*P*-value 0.005 and 0.003 for PEO1CarbR and PEO1CisR, respectively using a one-tailed paired test) and concomitant increase in S-phase: *P*-value 0.03 for PEO1CarbR, PEO1 0.26 and PEO1CisR 0.24, comparing control and treated cells using a one-tailed paired *t*-test). Statistical analysis of the sub-G1 populations of cells comparing untreated controls and following seliciclib treatment produced *P*-values that were for PEO1 0.05, PEO1CarbR 0.06 and for PEO1CisR 0.15 (using a one-tailed paired test). Thus, the S-phase change following treatment emerged as the best indicator of sensitivity to seliciclib. AnnexinV experiments using flow cytometry were also carried out and support the finding for a greater apoptotic response seen for seliciclib treated PEO1CarbR cells compared with PEO1 cells (data not shown), in line with the cytotoxicity data.

### Detection of methylation specific site in p57^kip2^ in carboplatin-sensitive and -resistant ovarian cancer cells

**Figure 4:**
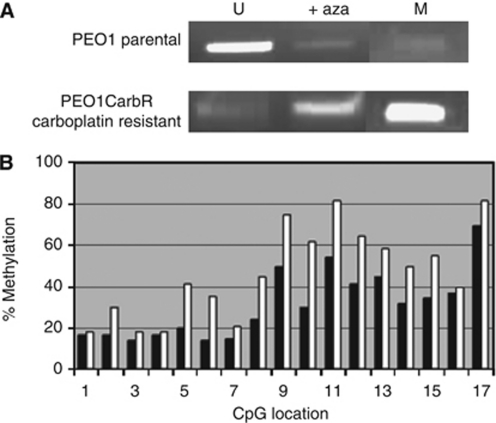
(**A**) Methylation specific PCR analysis for PEO1 and PEO1CarbR lines in the absence or presence of azacytidine (+aza) for the unmethylated portion (U) and for the methylated portion (M). Results shown are typical for those obtained in replicate experiments (*n*=4). (**B**) Pyrosequencing for detection and quantitation of methylation in the promoter region of the *p57*^*Kip2*^ gene with extent of methylation obtained from pyrosequencing analysis shown on the *y*-axis. Black bar indicates the parental PEO1 line; white bar indicates PEO1CarbR carboplatin resistant line.

Reversal of p57^*Kip2*^ silencing by 5-aza-2′deoxy-cytidine implies that methylation-dependent transcriptional silencing may underlie the reduced expression in PEO1CarboR. To directly address this possibility, we performed MSP analysis of the CpG island in the 5′ region of the *p57*^*KIP2*^ gene. Following PCR the resulting products were run on an agarose gel and visualised using ethidium bromide staining with transillumination. For the PEO1CarbR cells the level of methylated (M) p57^kip2^ was shown to be significant, with a very small band seen for the unmethylated (U) DNA, whereas for the PEO1 parental cells the predominant form of p57^kip2^ was seen to be of the unmethylated form. Treatment with aza-cytidine revealed a marked increase in the unmethylated form of p57^kip2^ for PEO1CarbR cells, Figure 4A. This corroborates the data obtained for protein expression as shown in the western blotting data, Figure 2A. Using the sensitive method of pyrosequencing, we were able to detect the exact level of methylation at each CpG location along the sequence positions 1361–1445, which matches well with the sequence we examined using MSP (1407–1520) (Figure 4B). The level of methylation for PEO1CarbR was shown to be significantly higher than for PEO1, *P*=0.026 and for PEO1CisR the levels of methylation were not significantly different from the parental cell line (*P*-value 0.285 using a paired *t*-test, data not shown).

### Silencing of p57^Kip2^ results in a decreased apoptotic response to platinum

**Figure 5:**
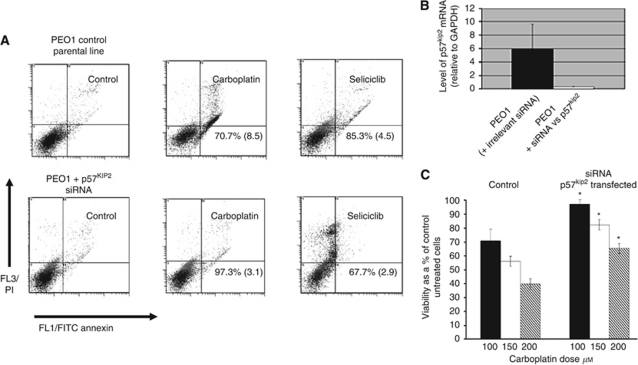
(**A**) Shows data obtained for PEO1 cells: control (using irrelevant siRNA, experimental control set) with carboplatin 100 *μ*M and seliciclib 25*μ*M for 48 h. PEO1 cells silenced with siRNA to p57^Kip2^ and drug treated as for control sample set. The lower left hand of the quadrant indicates the live cell population (i.e., PI and FITC negative), with various stages of apoptosis being observed in the others as upper left quadrant: PI positive (necrotic and/or end stage apoptosis), upper right PI- and FITC-positive mid/late stage apoptosis, lower right FITC only positive – early stage apoptosis. The percentage viability shown for each drug treatment indicates the viability expressed as a percentage of the control cells (considered as 100%). Numbers are shown as the mean and s.d. for three repeat experiments. *P*-value obtained for comparison of control seliciclib treated cells *versus* p57^kip2^ transfected seliciclib treated cells was 0.0085 (using a paired *t*-test). (**B**) Indicates efficient knockdown of P57^KIP2^ following siRNA transfection assessed using RT–PCR. (**C**) Flow cytometry data obtained using the Annexin assay (as in 5**A**) with bar graph data depicting viability (obtained from the Annexin V/PI-negative readout) expressed as a percentage of the control untreated cells for treatment with carboplatin at 100, 150 and 200 *μ*M. ^*^ A paired *t*-test gave *P*-values of 0.007, 0.012 and 0.007 for 100, 150 and 200 *μ*M, respectively, when comparing control (irrelevant siRNA) with corresponding result obtained for siRNA p57^kip2^ transfected cells.

Data shown in Figure 5A indicate the changes in apoptotic effects seen in at 48 h post-treatment in drug treated EOC cells following silencing with siRNA to p57^Kip2^. All experiments were carried out at least three times on PEO1 parental cell lines (not shown). Statistically significant decrease in apoptotic responses to carboplatin and increase in apoptotic response to seliciclib were seen in silenced *versus* control cells, see the legend to Figure 5A, which indicates cells in the viable region of the quadrant plot for the Annexin assay (lower left), expressed as a percentage of the untreated control cell viability (considered as 100%). Figure 5C shows the consistent lack of apoptosis induction for the p57^kip2^-silenced cells over the dose range of 100–200 *μ*M carboplatin treatment for 48 h. Comparison of control and transfected drug treated cell viabilities was carried out using the paired *t*-test (indicated in the figure legend). Similar experiments were carried out on SKOV-3 parental cells and the effects of p57^kip2^ silencing gave very similar (statistically significant) shifts in sensitivities to carboplatin and to seliciclib as seen for transfected PEO1 cells (data not shown).

### Clinical samples of EOC showed downregulation of p57^Kip2^ mRNA which was associated with worse outcome

**Figure 6:**
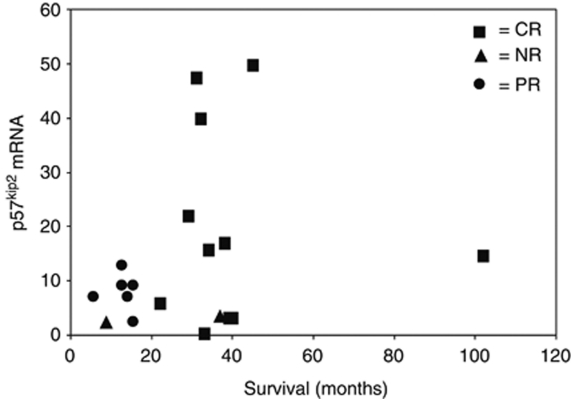
Scatterplot of mRNA p57^kip2^ expression levels plotted against length of survival showing association with differences in response to treatment as indicated by symbols. A significant difference between responses (CR *versus* PR and NR combined) for p57^kip2^ level, *P*=0.034 using a one-way ANOVA.

Statistical analysis of the data obtained from the qPCR analysis of RNA obtained from surgical samples of ovarian cancer showed a significant increase in survival for those cases where there was a CR to platinum-based chemotherapy compared with the other cases. A one-way ANOVA analysis for comparing p57^Kip2^ mRNA expression with a CR compared with partial and non-responders combined within the entire data set, which includes all the histological types, showed a significant difference: *P*-value of 0.034, Figure 6. In the data set obtained there was a significant increase in survival time (measured in months and for all histological types) associated with response to therapy (*P*=0.002 using a one-way ANOVA (data not shown).

## Discussion

There is increasing evidence that acquired resistance to cytotoxic chemotherapeutic drugs is driven by epigenetic rather than genetic mechanisms (Glasspool *et al*, 2006). Moreover, anticancer drug resistance is probably better described as a polygenic condition/state whereby many factors may be operative in one patient. The inactivation of many genes with important functions, such as tumour suppressor genes, has shown that aberrant methylation has an important role in human carcinogenesis. The data we provide in the current study explore this further in terms of evolution of drug resistance in difficult-to-treat cancers – notably EOC.

Moreover, we propose that p57^Kip2^ has a pivotal role in the chemo-responsiveness of EOC particularly in relation to platinum containing regimens – specifically for carboplatin. Methylation of *p57*^*KIP2*^ has been implicated in a number of malignant conditions, for example, the lung, breast and malignant mesotheliomas (Kobatake *et al*, 2004). However, to our knowledge, the role of this gene in anticancer drug resistance has not been reported until now.

Our study has made use of a panel of EOC lines with resistance to carboplatin, but we also used a cisplatin-resistant counterpart in parts of the study. Our study clearly shows that the association of drug resistance with downregulation of the *p57*^*kip2*^ gene and collateral sensitivity to the CDK2 inhibitor seliciclib was seen exclusively for the carboplatin-resistant cell line in our panel. Notwithstanding, we see cross-resistance to carboplatin in the PEO1CisR cisplatin-resistant line – but this is in the absence of p57^kip2^ downregulation and without concomitant relative sensitivity to seliciclib. The development of resistance to cancer chemotherapeutic drugs can result in cell lines with mixed and complex resistance phenotypes – even using the same class of chemotherapy drug as an inducing agent, such as platinum agents (Song *et al*, 2004).

A study by Vlachos *et al* (2007) indicated the importance of p57^Kip2^ in the commitment of drug-treated HeLa cells to drug-induced apoptosis and lends support to the role of this CDKI in response to anticancer drug therapy. Using an inducing transfection system it was shown that selective expression of *p57*^*KIP2*^sensitised cancer cells to the mitochondrial pathway of apoptosis following treatment with cisplatin, etoposide and staurosporine. Interestingly, the mechanism was shown to operate separately from any activity of p57^Kip2^ relating to its CDK inhibitory activity (although the sensitivity to seliciclib, at least in this system, suggests a CDK-dependent mechanism). Thus, this study corroborates the findings of the current study that equates a reduced expression of p57^Kip2^ with a more chemoresistant phenotype. Indeed, we did see a reduced sensitivity of *p57*^*kip2*^-silenced cells to the effects of cisplatin, as well as to carboplatin (data not shown).

Our findings that ovarian cancer cells with silenced p57^Kip2^ are more sensitive to the effects of the CDKI seliciclib are in keeping with the findings of Ma and Cress (2007). MDA-MB-231 cells deficient in p57^Kip2^ (using shRNA) were significantly more sensitive to the effects of the CDK2 inhibitor BMS-387032 in a similar manner to that seen for PEO1CarbR cells treated with seliciclib. In the same study it was shown that exogenous expression of p57^Kip2^ cells led to a decreased fraction of cells in the S-phase and repression of E2F1 transcription following treatment with BMS-387032. This is in line with our findings that p57^Kip2^ deficient PEO1CarbR cells are sensitised to the effects of seliciclib as shown by an enhanced apoptotic response and cytotoxicity.

The CDKIs have been implicated in the pathogenesis of ovarian cancer – with the expression of p27^Kip1^, but not p21^Waf1^ being associated with response to chemotherapy. Masciullo *et al* (1999) showed that p27^Kip1^ positivity was a strong predictor of survival, and was strongly associated with response to chemotherapy in those patients who were optimally debulked at the time of their first surgery. However, the expression of p21^Waf1^ was not associated with response to platinum agents or other chemotherapy in a group of 120 patients with EOC (Levesque *et al*, 2000). The present study shows *in vitro* evidence that measurement of p57^Kip2^ may be a useful prognostic indicator in EOC.

As well as genes such as methylation of *p57*^*Kip2*^, it is likely that many other events will contribute to the process of drug resistance in a given case of EOC. This is in line with studies published by other groups that have used clinical biopsies of EOC to monitor changes in CpG islands as a marker of disease progression (Wei *et al*, 2002). Serum or plasma from cancer patients often contains identical DNA changes to those present in the tumour and can thus provide a means for monitoring epigenetic changes within an individual tumour in a surrogate sample. We have recently completed a study involving matched blood and tissue from patients with EOC who were clearly defined into two cohorts of either relapsing or non-relapsing. We have gone on to measure the serum and plasma DNA for another gene – associated with the polo-like kinases and G_2_-checkpoint control – in those patients and have seen a statistically significant association between methylation status and relapse (Syed *et al*, 2011). As a continuation of the study presented herein our future studies involving p57^Kip2^ will involve measurement of methylated *p57*^*KIP2*^ DNA in tumour tissue and matched serum from a large cohort (>200) of ovarian cancer patients. We therefore plan to explore the utility of methylated *p57*^*KIP2*^ DNA as a prognostic indicator in ovarian cancer. Moreover, our data suggest that p57^Kip2^ could be used as a marker of sensitivity to the cell-cycle inhibitor seliciclib.

In conclusion, we present data that contribute to our understanding of drug resistant EOC and support the notion that this process can be explained on the basis of epigenetic modification, involving the CDKI p57^Kip2^. Moreover, our approach will help in the identification and evaluation of new prognostic markers for the management of EOC.
